# Effects of High-Intensity Inspiratory Muscle Training Associated with Aerobic Exercise in Patients Undergoing CABG: Randomized Clinical Trial

**DOI:** 10.21470/1678-9741-2018-0053

**Published:** 2018

**Authors:** Aline Paula Miozzo, Cinara Stein, Miriam Zago Marcolino, Isadora Rebolho Sisto, Melina Hauck, Christian Corrêa Coronel, Rodrigo Della Méa Plentz

**Affiliations:** 1 Universidade Federal de Ciências da Saúde de Porto Alegre, Porto Alegre, RS, Brazil.; 2 Instituto de Cardiologia, Porto Alegre, RS, Brazil.

**Keywords:** Coronary Artery Bypass, Cardiac Rehabilitation, Quality of Life, Exercise Therapy, Physical Therapy Modalities, Breathing Exercises, Muscle Strength/Physiology, Oxygen Consumption/Physiology, Respiratory Muscles/Physiopathology

## Abstract

**Objective:**

Evaluate the interaction between high-intensity inspiratory muscle training (IMT) and aerobic exercise on physical capacity, respiratory muscle strength, peripheral muscle strength, and quality of life of patients who underwent coronary artery bypass grafting (CABG).

**Methods:**

Twenty-four patients underwent CABG were randomized into two groups. During 36 sessions, one group received IMT associated with aerobic exercise and the other group received only aerobic exercise. Primary outcome was the distance in the six-minute walk distance (6MWD) test. Secondary outcomes included respiratory muscle strength, peripheral muscle strength, and quality of life. Measures were taken at the baseline, at the 12^th^ session, the 24^th^ session, and 36^th^ session.

**Results:**

Baseline characteristics were similar between the groups. There was no statistically significant difference between the two groups in any outcome [6MWD - *P*=0.935; peak oxygen consumption (PeakVO_2_) - *P*=0.853; maximal inspiratory pressure (MIP) - *P*=0.243; maximal expiratory pressure (MEP) - *P*=0.268; sitting-rising test (SRT) - *P*=0.212], but there was interaction in MIP (*P*=0.000) and all outcomes improved in the two groups (6MWD - *P*=0.000; PeakVO_2_ - *P*=0.000; MIP - *P*=0.000; MEP - *P*=0.000; SRT - *P*=0.000).

**Conclusion:**

There was an improvement of all outcomes in both groups, but IMT was not able to provide additional benefits. The use of this combination should be used with caution to not generate higher costs in the rehabilitation process of these patients.

**Table:** 

Abbreviations, acronyms & symbols				
6MWD	= Six-minute walk distance		IMT	= Inspiratory muscle training
CABG	= Coronary artery bypass grafting		MEP	= Maximal expiratory pressure
CHF	= Chronic heart failure		MIP	= Maximal inspiratory pressure
CI	= Confidence intervals		Peak VO_2_	= Peak oxygen consumption
CVD	= Cardiovascular diseases		SE	= Standard error
GAE-	= Aerobic exercise group		SRT	= Sitting-rising test
GAE+IMT	= Aerobic exercise + inspiratory muscle training group		WHO	= World Health Organization

## INTRODUCTION

Cardiovascular diseases (CVD) are expanding worldwide and according to the World Health Organization (WHO), 17.5 million people die each year from CVD, with 75% of these deaths occurring in low and middle income countries^[1]^. Coronary artery bypass grafting (CABG) aims to increase the life expectancy of patients with improved quality of life, relief of myocardial ischemia symptoms, and improvement of ventricular function^[2]^, but there are still high rates of complications^[3]^.

Aerobic exercise training shows a cardiovascular, skeletal muscle, endurance, pulmonary function, inflammatory profile, depression and stress symptoms, and quality of life improvement, as well as improving significant outcomes such as mortality^[4-6]^. Recent systematic reviews have shown that exercise training programs are capable of reducing mortality, reducing hospitalizations, and improving the quality of life of patients with coronary artery disease, chronic heart failure (CHF) and patients who have undergone CABG^[7-10]^.

For the treatment of patients with poor inspiratory muscle performance, with symptoms of dyspnea, low exercise tolerance, and low functional status^[11]^, inspiratory muscle training (IMT) is presented as a strategy of great value. In patients with CHF, low to moderate intensity IMT (maximum 30% of the maximal inspiratory pressure) led to a significant improvement in peak oxygen consumption (PeakVO_2_), functional capacity, and respiratory muscle strength, in addition to improving quality of life^[12-14]^.

High-intensity IMT [60% or more of the maximal inspiratory pressure (MIP)] has already been investigated, as in randomized clinical trials which showed that the use of this modality for four weeks in patients with CHF significantly improved respiratory muscle strength and percentage variation of resistance in these patients^[15]^. When associated with aerobic exercise, high-intensity IMT presented significant improvement in MIP, quality of life, dyspnea, and C-reactive protein^[16]^.

The effects of aerobic exercise and IMT on improved physical and clinical conditions of CVD patients are well established, however, no study has tested the additional effects of IMT associated with aerobic exercise in an attempt to minimize the side effects of cardiac surgery. Therefore, the objective of this study was to verify the effects of high-intensity IMT associated with aerobic exercise in functional capacity, respiratory muscle strength, peripheral muscle strength and quality of life of patients who underwent CABG.

## METHODS

### Design and Participants

This study was a randomized controlled clinical trial conducted between September 2015 and December 2016. Adults who underwent CABG were randomly assigned to perform aerobic exercise (GAE) or to perform aerobic exercise associated with high-intensity IMT (GAE+IMT) over a period of 3 months. Eligible participants were randomized using a computer-generated list of random numbers that had been prepared using a randomization model blocked by a researcher without involvement in the study. The assignment sequence was hidden in sealed, opaque, and sequentially numbered envelopes. After completing the baseline assessments, the lead investigator opened the appropriate envelope and assigned the participants to the group.

The sample consisted of patients in the postoperative period of elective CABG at the Institute of Cardiology of Rio Grande do Sul, Porto Alegre, Brazil. Patients between the fourteenth and the thirtieth postoperative day, aged 30 to 70 years, were referred for cardiac rehabilitation with medical authorization. Patients with decompensated CHF and presence of comorbidities, such as: unstable angina; moderate to severe respiratory disease; active infectious disease or febrile condition; disabling peripheral vascular disease; unstable ventricular arrhythmia and use of cardiac pacing were excluded. The sample size was calculated based on the study by Ghashghaei et al.^[17]^, which presented a variation of 98.15 meters in six-minute walk distance (6MWD) teste between groups with standard deviation of 87.89 meters and 66.75 meters with a power of 80% and level of significance of 5%. The calculated value included 10 patients in each group.

The entire intervention protocol was performed at the Cardiopulmonary and Metabolic Rehabilitation Center of the Institute of Cardiology of Rio Grande do Sul, in Porto Alegre, Brazil. Trained physiotherapists applied the protocols and the physiotherapists responsible for assessing the outcomes were unaware of the intervention.

### Ethical Considerations

This study was approved by the ethics and research committee of the Institute of Cardiology of Rio Grande do Sul, through protocol number 1.241.143 and was registered at Clinical Trials through number NCT02742350. All participants signed the informed consent.

### Procedures

Patients allocated to the GAE+IMT performed a protocol of high-intensity IMT followed by an aerobic exercise protocol. The high-intensity IMT protocol was performed with a linear pressure loading device (POWERbreathe Plus Resistance(r), SP, Brazil). IMT was performed for 12 weeks and the protocol consisted of five sets with 10 repetitions each until the 8^th^ week and progression of the number of sets (1 per week) and repetitions (10 to 12) from the 8^th^ to 12^th^ week. The overload was adjusted weekly by a revaluation of MIP starting with 50% of the MIP during the first two weeks, 60% of the MIP in the third and fourth weeks, 70% of the MIP in the fifth and sixth weeks, and 80% MIP from the seventh week until the end of the protocol.

The aerobic exercise protocol was performed for 12 weeks and divided into 3 phases: Phase 1 - 12 sessions with 50% to 60% of reserve PeakHR (maximal heart rate); Phase 2 - 12 sessions with 60% to 70% of the reserve PeakHR, and Phase 3 - 12 sessions with 70% to 80% of the reserve PeakHR. Exercise prescription was given through the ergometric test, where PeakHR and maximum oxygen consumption (PeakVO_2_) through repetitive effort with the Bruce protocol were obtained. The aerobic training had an average duration of 40 minutes.

Patients allocated to the GAE performed the same aerobic exercise protocol performed in the GAE+IMT, during the same 12 weeks. These patients did not perform the high-intensity IMT protocol.

### Outcomes

The evaluations were performed pre-intervention, at the 12^th^, 24^th^, and 36^th^ session. Except for the quality of life and ergometric test that were evaluated only pre and post-intervention.

Functional capacity was the primary outcome assessed through the 6MWD, which was performed according to the guidelines proposed by the American Thoracic Society^[18]^. The patients made their way down a thirty-meter corridor delimited by cones, encouraged by the evaluator every minute. Another functional capacity assessment was evaluated by the indirect measurement of PeakVO_2_ through the pre and post-intervention by ergometric test.

The circuit for the measurement of respiratory muscular strength, MIP and maximal expiratory pressure (MEP) was composed of an MVD 300 digital manometer (Microhard System, Globalmed, Porto Alegre, Brazil). MIP was measured from residual volume and MEP from total lung capacity^[19]^.

To evaluate the peripheral muscular strength, the sitting-rising test (SRT) was performed with the patient sitting in a 45 cm high chair, feet apart and supported on the floor with arms crossed against the chest. The patient was encouraged to stand up and return to a sitting position as many times as possible in 30 seconds, with the maximum number of repetitions recorded^[20]^.

In order to evaluate the impact of modifying some daily habits on quality of life and health maintenance, the patients participating in the study from both groups were invited to fill out a questionnaire on quality of life at the time of inclusion in the study and at the end of the study. The Brazilian Version of the Quality of Life Questionnaire - SF-36 was used.

### Statistical Analysis

Descriptive data are presented as mean and standard error (SE). Variations between interventions are reported as mean differences with 95% confidence intervals (95% CI). The distribution of variables was tested by Shapiro-Wilk normality test. In the statistical analysis, the Student's t-test was applied to compare means or two repeated measures. Effects of interventions were compared by generalized estimating equation model (group, time, and interaction), followed by Bonferroni *post-hoc* test. The categorical variables were evaluated by Fisher's exact test, and are expressed as proportions. The statistical analyses were performed using the SPSS 24, graphics were made by Graphpad Prism 5, and the level of significance was set at 0.05.

## RESULTS

**Fig. 1:**
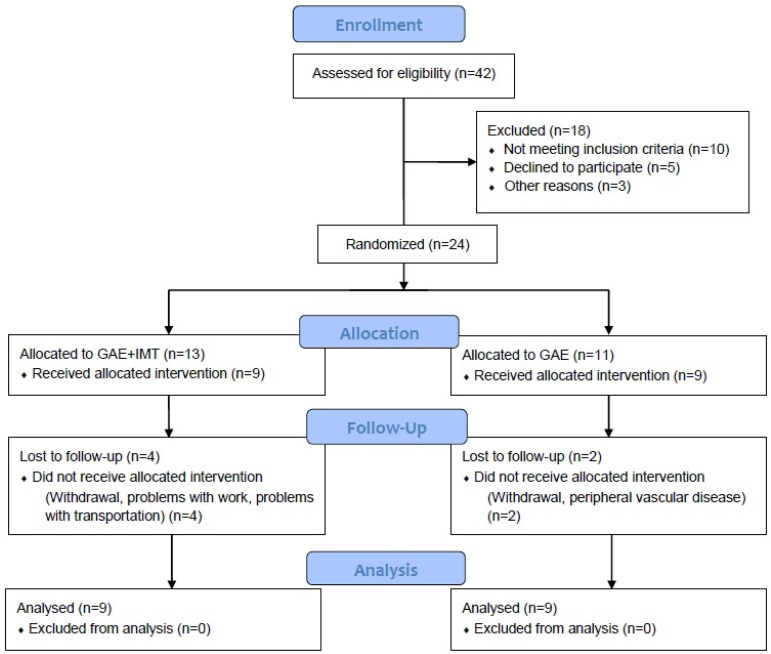
Flow chart of participant recruitment and study enrollment. GAE= aerobic exercise group; GAE+IMT= aerobic exercise + inspiratory muscle training group

The participant's flow through the study is summarized in Figure 1. A total of 42 potential volunteers were screened. 24 participants meeting the eligibility criteria were randomized to the GAE+IMT (n=13) and the GAE (n=11), and 6 did not finish the protocol. In the GAE+IMT: one patient was excluded due to discovery of peripheral arterial disease; one patient by blood glucose test, and two patients withdrew from the protocol. Two patients from the GAE gave up the protocol because of problems with work. The sample was considered homogeneous as the basic characteristics. Clinical characteristics were presented in Table 1.

**Table 1:** Baseline characteristics of participants.

	GAE (n=9)	GAE+IMT(n=9)	*P* value
Age (years)	57.4±8.54	57.6±7.9	0.95
Body mass index (kg/m^2^)	28.1±2.79	25.4±3.02	0.06
NYHA I (n)	9 (100%)	9 (100%)	1.00
Hypertension (n)	8 (88.9%)	7 (77.8%)	0.50
Dyslipidemia (n)	8 (88.9%)	9 (100%)	0.50
Statins (n)	8 (88.9%)	6 (66.7%)	0.29
Beta blockers (n)	4 (44.4%)	4 (44.4%)	0.68
Anticoagulants (n)	8 (88.9%)	4 (44.4%)	0.06
Antihypertensives (n)	6 (66.7%)	2 (22.2%)	0.07
6MWD (m)	545.5±31.11	543.3±25.45	0.95
MIP (cmH_2_O)	81.7±8.28	79.44±9.15	0.85
MEP (cmH_2_O)	114.3±11.73	111.9±9.86	0.87
SRT (n)	17.1±1.26	15.9±0.85	0.25

6MWD=six-minute walk distance; GAE=aerobic exercise group; GAE+IMT=aerobic exercise + inspiratory muscle training group; MEP=maximal expiratory pressure; MIP=maximal inspiratory pressure; NYHA=New York Heart Association; SRT=Sitting-rising test

**Fig. 2:**
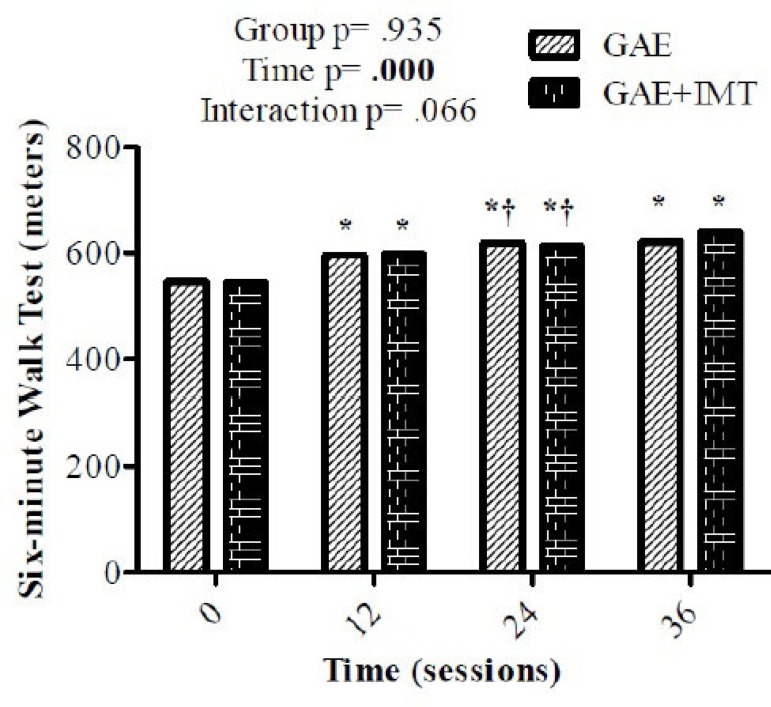
Six-minute walk distance (6MWD) Repeated measures Two-Way ANOVA followed by Bonferroni post-hoc test. GAE= aerobic exercise group; GAE+IMT= aerobic exercise + inspiratory muscle training group *versus 0 †versus 12; 24 X 36 = P=0.105

No significant difference was obtained in functional capacity with the 6MWD in any of the four moments when comparing groups (*P*=0.935). However, when we set time, significant difference was found in both groups in all times (*P*=0.000). This difference increases until time 24, but time 36 presents significant difference only when compared to time 0 and time 12 (24 *vs*. 36 = *P*=0.105) (Figure 2). The maximal oxygen consumption collected in the ergometric test showed similar results. There was no significant difference between the groups in the pre and post-intervention evaluation (Peak VO_2_, *P*=0.853), but there was improvement between the two moments within each group (Peak VO_2_, *P*=0.000) (Table 2).

**Table 2:** Peak VO_2_.

	Time 0	Time 12	Time 24	Time 36	Group	Time	Interaction
Group AE	17.1±1.26 (14.6-19.6)	19.5±1.58* (14.6-19.6)	21.9±1.65*† (18.6-25.13)	24.3±1.93*†‡ (20.5-28.1)	0.212	0.000	0.540
Group AE+IMT	15.9±0.85 (14.2-17.5)	18.3±0.78 (16.8-19.8)	19.2±1.2*† (16.8-21.6)	20.9±1.4*†‡ (18.1-23.7)			

GAE=aerobic exercise group; GAE+IMT=aerobic exercise + inspiratory muscle training group; PeakVO_2_=maximal oxygen consumption

* *versus* 0,

† *versus* 12 ,

‡ *versus* 24

**Fig. 3:**
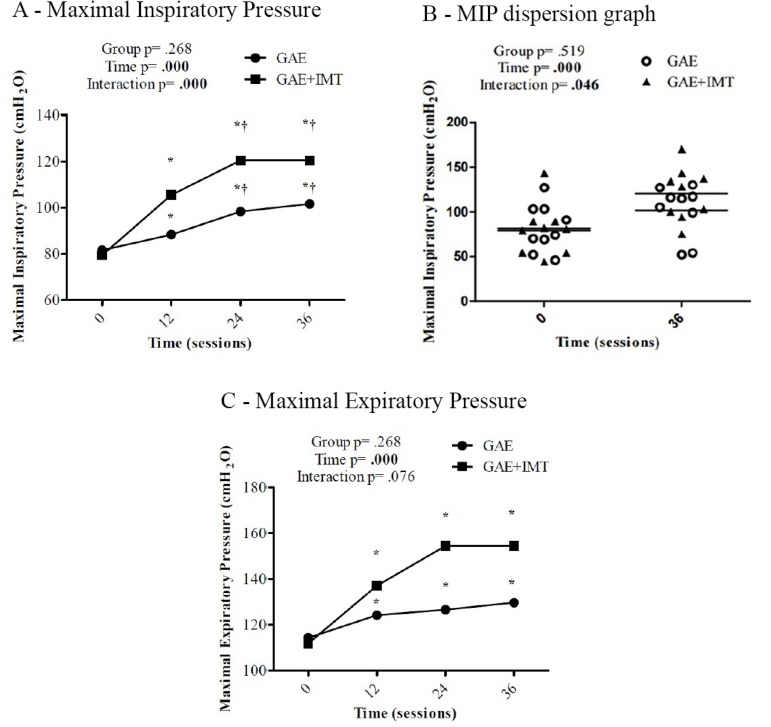
Respiratory Muscle Strength. A- Repeated measures Two-Way ANOVA followed by Bonferroni post-hoc test. B- Repeated measures Two-Way ANOVA followed by Bonferroni post-hoc test. C- Repeated measures Two-Way ANOVA followed by Bonferroni post-hoc test GAE= aerobic exercise group; GAE+IMT= aerobic exercise + inspiratory muscle training group *versus 0 †versus 12; MIP - 24 X 36 = P=1.000 C- *versus 0 †versus 12; MEP - 12 X 24 = P=0.501, 12 X 36 = P=0.269, 24 X 36 = P=1.000

Regarding respiratory muscle strength, when we compared the groups, there was no significant difference in MIP in any of the four moments (*P*=0.243). However, while setting the time, significant difference was found in both groups in all times (*P*=0.000). This difference increases until time 24, but time 36 presents significant difference only when compared to time 0 and time 12 (24 *vs*. 36 = *P*=1.000). When we set time, there was a significant interaction between the groups, always favoring the GAE+IMT group. This difference is most evident at time 24 with a strong tendency in GAE+IMT with a mean difference of 22.11±12.07 (GAE= 98.33; GAE+IMT=120,44; *P*=0.067) (Figure 3A). In dispersion measure (Figure 3B), we can see this interaction of MIP between moments 0 and 36 in both groups. Although the GAE + IMT group started lower, it reached higher values in manovacuometry. It is important to note that the difference between pre and post in the groups was approximately 20% in the GAE and 40% in the GAE+IMT and 20% between the groups. These values, when interpreted clinically, represent a significant gain and an additional effect of IMT in this population.

The results of the MEP did not present significant difference between the groups at any moment (*P*=0.268). When comparing the moments within each group, there were only differences at times 12, 24, and 36 when compared to moment 0 (12 *vs*. 24 = *P*=0.501; 12 *vs*. 36 = *P*=0.269; 24 *vs*. 36 = *P*=1.000) (Figure 3C).

About peripheral muscular strength regarding the SRT, when comparing the groups, no significant difference was obtained in any of the four moments (*P*=0.212). However, when we set time, there is significant difference at all times in both groups (*P*=0.000). This difference increases at time 12, time 24, and time 36 (Table 3).

**Table 3:** Peripheral muscular strength, sitting-rising test.

Peak VO_2_	Time 0	Time 36	Group	Time	Interaction
GAE	29.9±2.26 (25.46-34.32)	35.6± 1.96* (31.8-39.5)	0.853	0.000	0.660
GAE+IMT	29.5±0.73 (28.01-30.96)	36.6± 1.26* (34.2-39.13)			

GAE= aerobic exercise group; GAE+IMT= aerobic exercise + inspiratory muscle training group; PeakVO_2_= maximal oxygen consumption

* *versus* 0

Regarding the quality of life, the eight domains of the SF-36 questionnaire, there was no significant difference between the groups at any time pre and post-intervention. When we set the time, the domains general state (GAE+IMT pre-57.22±5.65 post-52,77±5,65; GAE pre-52.77±3.63 post-50±8.29; *P*=0.07), social aspects (GAE+IMT pre-43.05±12.67 post-44.38±6.65; GAE pre-48.61±7.51 post-51.38±14.58; *P*=0.21), and mental health (GAE+IMT pre-62.22±7.51 post-63.55±7.85; GAE pre-56.44±6.14 post-62.66±4.89; *P*=0.06) did not obtain a difference. The domains, functional capacity (GAE+IMT pre-61.11±15.76 post-81.66±15; GAE pre-61.11±13.86 post-88.33±7.9; *P*<0.000), physical aspects (GAE+IMT pre-0±0 post-52±47.5; GAE pre-8.33±2.77 post-75±39.52; *P*<0.000), pain (GAE+IMT pre-42.22±13.01 post-17.77±13.94; GAE pre-36.66±15 post-21.11±20.77; *P*<0.000), vitality (GAE+IMT pre-57.77±10.03 post-67.22±10.92; GAE pre-53.33±5.59 post-69.44±15.09; *P*<0.000), and emotional aspects (GAE+IMT pre-44.44±52.7 post-85.17±33.79; GAE pre-55.54±47.14 post-85.16±24.23; *P*=0.001) presented significant difference in both groups.

## DISCUSSION

This randomized clinical trial was the first study to associate the use of high-intensity IMT with aerobic exercise in patients who underwent CABG. The main findings of this study were a demonstration that there was no additional increase of the high-intensity IMT at the main endpoint, no test of the patients without a 6MWD and also in the secondary outcomes: Peak VO_2_, MEP, SRT and quality of life. However, there was a significant MIP interaction, showing additional benefit for the group that performed IMT.

These results are at odds with some studies in other populations. The use of IMT with low loads was effective in respiratory muscle strength, functional capacity, and quality of life in a systematic review of patients with chronic obstructive pulmonary disease^[21]^. In a systematic review of patients with CHF, IMT was able to improve Peak VO_2_, 6MWD, and MIP^[12-14]^. In CABG patients, IMT associated with aerobic exercise improved pre and post-intervention variation in MIP, Peak VO_2_, and quality of life^[22]^. The only study that involved the interaction of high-intensity IMT with aerobic exercise was performed in patients with CHF and found positive results in the improvement of the quality of life, but the results of the MIP were similar to ours, showing that there was only difference over time, without difference between the groups^[16]^.

The disagreement in the results can be justified by the difference in the number of cases, by the severity of the disease and by the type of intervention performed, since the greatest results are demonstrated in studies involving IMT alone without association with other interventions. The systematic review by Montemezzo et al.^[12]^ shows that the effect of IMT on 6MWD was only significant when training was performed on patients with previous inspiratory muscle weakness, which did not characterize the sample of this study.

The improvement in MIP observed in this study is justified by the principles of training: overload, in which the muscle must be requested at levels higher than usual so that muscle cells increase in size or functional capacity and specificity, where the training should be directed specifically to the muscle properties^[23]^. Many studies corroborate this finding both in the use of low-load IMT as in the studies of Gosselink et al.^[21]^, with chronic obstructive pulmonary disease, or of Hermes et al.^[22]^, postoperative node of CABG, or in the systematic reviews of patients with CHF of Montemezzo et al.^[12]^, Plentz et al.^[13]^, and Smart et al.^[14]^, as well as in high-intensity use observed in the study by Adamopoulos et al.^16^.

It is important to highlight that the groups obtained significant improvements in all the outcomes studied, showing that both trainings are alternatives that can be used to recover patients who underwent CABG. However, the use of technology without additional gains makes intervention more costly to the patient and health systems, and should be used with caution.

### Limitation

The study presents some limitations that could have improved the effect of the intervention. Although the sample number is in accordance with a previous statistical calculation, the increase in the number of the sample could increase the power of the results. In addition, there was a lack of blinding of therapists and patients as well as the lack of a non-intervention group in order to compare one training with another. That being said, new studies can be carried out in an attempt to improve the hypothesis.

## CONCLUSION

This was the first study to associate the use of high-intensity IMT with aerobic exercise in the postoperative period of CABG. There was an improvement of all outcomes in both groups, but high-intensity IMT was not able to provide additional benefit in most of the outcomes, being observed only in inspiratory muscle strength. Therefore, the use of this combination should be used with caution so as to not generate higher costs in the rehabilitation process of these patients.

**Table:** 

Authors' roles & responsibilities	
APM	Substantial contributions to the conception or design of the work; or the acquisition, analysis, or interpretation of data for the work; drafting the work or revising it critically for important intellectual content; final approval of the version to be published
CS	Substantial contributions to the conception or design of the work; or the acquisition, analysis, or interpretation of data for the work; drafting the work or revising it critically for important intellectual content; final approval of the version to be published
MZM	Substantial contributions to the conception or design of the work; or the acquisition, analysis, or interpretation of data for the work; drafting the work or revising it critically for important intellectual content; final approval of the version to be published
IRS	Substantial contributions to the conception or design of the work; or the acquisition, analysis, or interpretation of data for the work; drafting the work or revising it critically for important intellectual content; final approval of the version to be published
MH	Substantial contributions to the conception or design of the work; or the acquisition, analysis, or interpretation of data for the work; drafting the work or revising it critically for important intellectual content; final approval of the version to be published
CCC	Drafting the work or revising it critically for important intellectual content; final approval of the version to be published
RDMP	Substantial contributions to the conception or design of the work; or the acquisition, analysis, or interpretation of data for the work; final approval of the version to be published
